# Hardness Recognition of Robotic Forearm Based on Semi-supervised Generative Adversarial Networks

**DOI:** 10.3389/fnbot.2019.00073

**Published:** 2019-09-06

**Authors:** Xiaoliang Qian, Erkai Li, Jianwei Zhang, Su-Na Zhao, Qing-E Wu, Huanlong Zhang, Wei Wang, Yuanyuan Wu

**Affiliations:** School of Electrical and Information Engineering, Zhengzhou University of Light Industry, Zhengzhou, China

**Keywords:** tactile sensing, hardness recognition, deep learning, semi-supervised, generative adversarial networks

## Abstract

The hardness recognition is of great significance to tactile sensing and robotic control. The hardness recognition methods based on deep learning have demonstrated a good performance, however, a huge amount of manually labeled samples which require lots of time and labor costs are necessary for the training of deep neural networks. In order to alleviate this problem, a semi-supervised generative adversarial network (GAN) which requires less manually labeled samples is proposed in this paper. First of all, a large number of unlabeled samples are made use of through the unsupervised training of GAN, which is used to provide a good initial state to the following model. Afterwards, the manually labeled samples corresponding to each hardness level are individually used to train the GAN, of which the architecture and initial parameter values are inherited from the unsupervised GAN, and augmented by the generator of trained GAN. Finally, the hardness recognition network (HRN), of which the main architecture and initial parameter values are inherited from the discriminator of unsupervised GAN, is pretrained by a large number of augmented labeled samples and fine-tuned by manually labeled samples. The hardness recognition result can be obtained online by importing the tactile data captured by the robotic forearm into the trained HRN. The experimental results demonstrate that the proposed method can significantly save the manual labeling work while providing an excellent recognition precision for hardness recognition.

## Introduction

The tactile sensing is an important direction in artificial intelligence (AI) research, and is especially useful for the robotic arms to mimic human hands in grasping and other movements (Xiaonan, 2011). In order to achieve human-like robotic arms, two tactile recognition studies need to be carried out. One study focuses on using visual and tactile data together to recognize the object (Gao et al., 2016; Falco et al., 2017; Levine et al., 2017; Liu et al., 2017). The other study focuses on using the tactile sensing data to obtain the physical parameters of the object, such as texture, hardness (Ahmadi et al., 2010; Kaboli et al., 2014; Hoelscher et al., 2015; Yamazaki et al., 2016). The hardness is one typical parameter essential to the grasping force control for the robotic forearm (Schill et al., 2012; Huang et al., 2013; Lichao, 2016), which is the focus of this paper. The existing hardness recognition methods can be broadly classified into two categories: (1) non-machine learning based methods, (2) machine learning based methods.

A majority of previous hardness recognition methods can be classified into the first category. Huang et al. (2012) used the pressure data and the grasping position of the robotic hand to test the hardness of the object. Yussof et al. (2008) let the robotic hand touch the object several times using various small forces, obtaining the hardness based on the force feedback. Boonvisut and Cavusoglu (2014) used the shape change of the object to recognize the hardness. The non-machine learning based methods usually did not use the complex algorithms but require sophisticated hardware and complex testing procedures.

The hardness recognition works belonging to the second category can be further classified into two types according to whether or not the deep learning techniques are employed. The first type is based on traditional machine learning approaches. Chu et al. (2015) used BioTac sensors to obtain tactile data. Then, the hidden markov model (HMM) is used to extract the feature vectors of tactile data. Finally, the support vector machine (SVM) is trained and used to recognize the hardness. Other methods based on traditional machine learning include: decision tree based method (Bandyopadhyaya et al., 2014), k-nearest neighbors (KNN) based method (Drimus et al., 2011), and SVM based method (Kaboli et al., 2014), etc. Recently, the deep learning technique has made great progress and has been successfully applied in many fields (Han et al., 2013, 2015; Wu et al., 2015, 2017; Li et al., 2016; Zhang et al., 2017; Hou et al., 2018). Some hardness recognition works based on deep learning included are as follows: Yuan et al. (2017) used a GelSight sensor to obtain the tactile data sequence, and adopted the long short-term memory (LSTM) algorithm to recognize the hardness of the object. Bhattacharjee et al. (2018) also used LSTM to process the time-variant tactile sensing data to classify the hardness of the object. The visual and tactile features are extracted by convolution neural network (CNN), and combined for hardness recognition (Gao et al., 2016).

The deep learning based methods have shown their superiority among aforementioned methods, however, the existing deep learning based methods require a large number of manually labeled samples which need significant time and labor costs. In order to alleviate this problem, a hardness recognition method based on semi-supervised GAN is proposed in this paper. The proposed method not only makes use of a large number of unlabeled samples, but also augments the labeled samples.

**Figure 1:**
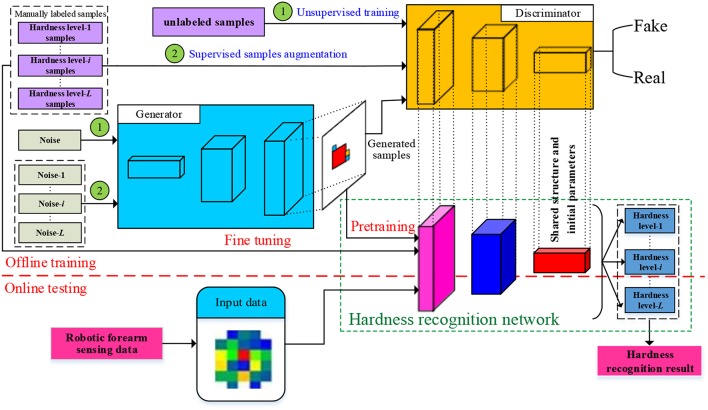
Framework of proposed method.

The framework of the proposed method is shown in Figure 1. In the training stage of GAN, first of all a large number of unlabeled samples are used to train a GAN which is denoted as USTGAN (unsupervised training GAN). Secondly, the manually labeled samples corresponding to each hardness level are separately used to train *L* (number of hardness level) GANs which are denoted as STGAN (supervised training GAN), where the architecture and initial parameter values of STGAN are inherited from USTGAN. The *L* levels manually labeled samples are separately augmented by the *L* trained STGANs. In the training stage of HRN, a large number of augmented samples are used to pretrain the HRN of which the main architecture and initial parameter values are inherited from the discriminator of USTGAN, and then the manually labeled samples are used to fine-tune the HRN. In the testing stage, the captured tactile data obtained by the robotic forearm are directly imported into the HRN to obtain the hardness recognition results. The DeLiGAN (Gurumurthy et al., 2017) is used as the GAN model in this paper because it requires less labeled samples. It's worth noting that the other published GAN models, such as GM-GAN (Ben-Yosef and Weinshall, 2018), can also be adopted and the selection of GAN model is not the focus of this paper.

As a matter of fact, some semi-supervised GANs have been proposed (Odena, 2016; Salimans et al., 2016), however, the role of the proposed semi-supervised GAN is different in previous methods. The discriminator of previous semi-supervised GANs is used as the classifier which outputs *L*+1 probabilities (*L* probabilities for the *L* real classes and one probability for the fake classes), therefore, the trained discriminator can be directly used to recognize the hardness level. However, the proposed semi-supervised GAN is used to augment the manually labeled samples rather than classification of hardness level. As mentioned before, after the unsupervised training of USTGAN which is used to provide initial model parameters to STGAN and HRN, *L* level manually labeled samples are used to train *L* STGANs and are subsequently augmented by the *L* trained generators of STGANs. As a matter of fact, the discriminator of STGAN is the traditional real-fake binary classifier which is not used for the classification of hardness level, and the classification of hardness level is implemented by the HRN which outputs *L* probabilities. A large number of samples augmented by STGANs are used to pretrain the HRN, which is the key contribution of STGAN.

The major contributions of the proposed method can be summarized as follows:

1. A semi-supervised scheme is proposed for hardness recognition in order to save the time and labor cost of human labeling. A large number of unlabeled samples are made use of through the unsupervised training of USTGAN, of which the main architecture and parameter values are shared with the following STGAN and HRN, and the manually labeled samples are used to train STGAN and automatically augmented by the trained STGAN.
2. A HRN of which the main architecture and initial parameter values are inherited from the discriminator of USTGAN is employed to recognize the hardness level of objects touched by robotic forearm online. The large amount of augmented samples and manually labeled samples are separately used to pretrain and fine-tune the HRN.

## Proposed Method

The proposed method can be classified into three steps. Firstly, the USTGAN is trained by a large number of unlabeled samples in order to provide a good initial state to the following STGAN and HRN. Secondly, *L* STGANs of which the generators are used to augment the labeled samples are individually trained by *L*-level manually labeled samples. Finally, the HRN which is pretrained by augmented samples and fine-tuned by manually labeled samples is employed to recognize the hardness level of captured tactile data obtained from the robotic forearm.

As mentioned before, the architecture of DeLiGAN is adopted by STGAN and USTGAN in this paper, therefore, some of the details of DeLiGAN will be presented in the following section for the completeness of description. The details of above three steps are given as follows.

### Unsupervised Training of USTGAN

In order to alleviate the shortage of labeled samples, the large number of unlabeled samples are made use of by sharing the architecture and parameter values of trained USTGAN with the following STGAN and HRN.

The architecture and training scheme of USTGAN is similar to DeLiGAN, the only difference is the training samples. Therefore, the details of the training scheme of USTGAN is no longer presented in this section.

### Labeled Samples Augmentation Based on STGAN

The architecture of STGAN is the same as USTGAN and the initial parameter values are also given by the trained USTGAN, however, the training of STGAN is supervised. As shown in Figure 1, the probability density of input noises of *i*th STGAN which is used to augment the *i*th hardness level samples is defined as follows (Gurumurthy et al., 2017):

(1) $$\begin{array}{l} \begin{array}{c} p\left(z_{i}\right)=\overset{K}{\underset{k=1}{\sum }}\pi_{i}^{k}N\left(z_{i}|\mu_{i}^{k},\Sigma_{i}^{k}\right),\;i\in \left[1,L\right]\\ s.t.\;\overset{K}{\underset{k=1}{\sum }}\pi_{i}^{k}=1 \end{array} \end{array}$$

where, $z_{i}\in R^{n}$ denotes the input noises corresponding to *i*th hardness level, *n* is the dimension of the input noises, *K* denotes the number of Gaussian component contained in GMM, N(·) denotes the Gaussian distribution, $\mu_{i}^{k}\in R^{n}$ and $\Sigma_{i}^{k}\in R^{n\times n}$ denotes the mean vector and covariance matrix of the *k*th Gaussian component of *z*_*i*_, respectively, $\pi_{i}^{k}$ denotes the weight of *k*th Gaussian component of *z*_*i*_, which can be quantified by ratio of the number of noisy signals generated from *k*th Gaussian component to all noisy signals.

To obtain the *z*_*i*_, the “reparameterization trick” introduced by Kingma and Welling (2014) is employed to sample from each Gaussian component. We assume that $\Sigma_{i}^{k}$ is a diagonal covariance matrix which is denoted as $\Sigma_{i}^{k}$ = diag ($\sigma_{i}^{k_{1}}\;\sigma_{i}^{k_{2}}\cdots \sigma_{i}^{kn}$), then the input noise derived from the *k*th Gaussian component which is denoted as $z_{i}^{k}\in R^{n}$ can be obtained by:

(2) $$\begin{array}{l} z_{i}^{k}=\mu_{i}^{k}+\sigma_{i}^{k}\eta \\ \sigma_{i}^{k}=\left[\sigma_{i}^{k_{1}}\;\sigma_{i}^{k_{2}}\cdots \sigma_{i}^{kn}\right]\in R^{n}\\ s.t.\eta \sim N\left(0,1\right) \end{array}$$

where, η is an auxiliary noise variable following normal distribution, $\sigma_{i}^{k}$ denotes the diagonal elements of $\Sigma_{i}^{k}$. As shown in Equation (2), obtaining the $z_{i}^{k}$ will translates to sampling η~N(0,1) if the values of $\mu_{i}^{k}$ and $\sigma_{i}^{k}$ are obtained. Finally, the *z*_*i*_ can be obtained by repeating the above processing of each $z_{i}^{k}$ *k* ∈ [1, *K*].

Obviously, the values of $\mu_{i}^{k}$ and $\sigma_{i}^{k}$ should be determined in order to obtain the input noise. The $\mu_{i}^{k}$ and $\sigma_{i}^{k}$ are learned along with the training of *i*th STGAN of which the details are as follows.

#### Training of STGAN

A total of *L* STGANs are trained by *L* level manually labeled samples, respectively. The loss function of discriminator of *i*th STGAN is as follows (Goodfellow et al., 2014; Radford et al., 2016):

(3) $$\begin{array}{l} \underset{\theta^{D}}{\min}\left(\underset{z_{i}\sim p\left(z_{i}\right)}{\sum }\log\left(D\left(G\left(z_{i},\theta_{i}^{G}\right),\theta_{i}^{D}\right)\right)-\underset{x_{i}\sim p_{data}\left(x_{i}\right)}{\sum }\log D\left(x_{i},\theta_{i}^{D}\right)\right) \end{array}$$

where, $\theta_{i}^{D}$ and $\theta_{i}^{G}$ denote the model parameters of discriminator and generator of *i*th STGAN, *x*_*i*_ denotes the manually labeled samples of *i*th hardness level, *p*_*data*_(*x*_*i*_) denotes the probability density distribution of *x*_*i*_,*G*(·) denotes the samples generated from *z*_*i*_, *D*(·) ∈ [0, 1] denotes the probability that the input samples belong to the real labeled samples. As shown in Equation (3), $\theta_{i}^{G}$ is regarded as constants when discriminator is trained using Equation (3).

The loss function of generator is as follows (Goodfellow et al., 2014; Radford et al., 2016):

(4) $$\begin{array}{l} \underset{\theta^{G}}{\max}\left(\underset{z_{i}\sim p\left(z_{i}\right)}{\sum }\log\left(D\left(G\left(z_{i},\theta_{i}^{G}\right),\theta_{i}^{D}\right)\right)\right) \end{array}$$

Similarly, $\theta_{i}^{D}$ is regarded as constants when the generator is trained using Equation (4).

As shown in Equation (2), $z_{i}=\left[z_{i}^{1}\;\cdots z_{i}^{k}\cdots z_{i}^{K}\right]$ is the function of $\mu_{i}^{k}$ and $\sigma_{i}^{k}$, therefore, the $\mu_{i}^{k}$ and $\sigma_{i}^{k}$ will be trained simultaneously along with $\theta_{i}^{G}$. According to Equation (2), the Equations (3, 4) can be respectively reformulated as:

(5) $$\begin{array}{l} \underset{\theta^{D}}{\min}(\sum_{\begin{array}{c} \eta \sim \;N(0,1)\\ k\in [1,K] \end{array}}\log(D(G(\eta ,\theta_{i}^{G},\mu_{i}^{k},\sigma_{i}^{k}),\theta_{i}^{D}))\\ \;-\sum_{x_{i}\sim \;p_{data}(x_{i})}\log D(x_{i},\theta_{i}^{D})) \end{array}$$

(6) $$\underset{\theta^{G},\mu_{i}^{k},\sigma_{i}^{k}}{\max}\left(\sum_{\begin{array}{c} \eta \sim \;N(0,1)\\ k\in [1,K] \end{array}}\log(D(G(\eta ,\theta_{i}^{G},\mu_{i}^{k},\sigma_{i}^{k}),\theta_{i}^{D}))\right)$$

It's worth noting that generator tries to decrease the $\sigma_{i}^{k}$ in order to obtain more noisy signals from the high probability regions which are around the $\mu_{i}^{k}$, consequently, the $\sigma_{i}^{k}$ will collapse to zero. Therefore, a L_2_ penalty terms is added to the loss function of generator in order to prevent this from happening:

(7) $$\begin{array}{l} \underset{\theta^{G},\mu_{i}^{k},\sigma_{i}^{k}}{\max}\left(\underset{\begin{array}{c} \eta \sim N\left(0,1\right)\\ k\in \left[1,K\right] \end{array}}{\sum }\log\left(D\left(G\left(\eta ,\theta_{i}^{G},\mu_{i}^{k},\sigma_{i}^{k}\right),\theta_{i}^{D}\right)\right)-\lambda {\left(1-\sigma_{i}^{k}\right)}^{2}\right) \end{array}$$

Finally, the discriminator and generator of STGAN is trained alternatively according to Equations (5) and (7) for obtaining the $\theta_{i}^{D}$, $\theta_{i}^{G}$, $\mu_{i}^{k}$, and $\sigma_{i}^{k}$.

#### Samples Generation

According to Equation (2), $N_{i}^{k}$ noise signals can be generated from the *k*th Gaussian component when $\mu_{i}^{k}$ and $\sigma_{i}^{k}$ have been learned in the last section. Subsequently, the $N_{i}^{k}$ generated samples are obtained by sending the $N_{i}^{k}$ noise signals to the generator of *i*th STGAN. Finally, the $N_{i}=\Sigma_{k=1}^{K}N_{i}^{k}$ generated samples corresponding to the *i*th hardness level are obtained and denoted as *gx*_*i*_.

### Hardness Recognition

As shown in Figure 1, the architecture of HRN is the same as the discriminator of USTGAN except for the classification layer. Specifically, the classification layer of USTGAN and HRN is the real-fake binary classifier and *L*-class softmax classifier, respectively. The HRN is firstly pretrained using *GX* and is subsequently fine-tuned using *X*, where, $GX={\left\{gx_{i}\right\}}_{i=1}^{L}$ and $X={\left\{x_{i}\right\}}_{i=1}^{L}$ denote the assemble of *gx*_*i*_ and *x*_*i*_, respectively.

As mentioned before, the initial model parameters of HRN are inherited from the discriminator of trained USTGAN. The rationality of the parameters sharing can be analyzed in terms of loss function. In fact, the discriminator of USTGAN can be regarded as a combination of real-fake classifier and CNN which is used to extract the deep feature of generated data and real data. Consequently, the loss function of discriminator of USTGAN can be reformulated as:

(8) $$\begin{array}{l} \underset{\beta^{D}}{\min}(\sum_{\varepsilon \sim \;p(\varepsilon )}\log(P(f(\varepsilon ,\beta^{G})\in real|\beta^{D}))\\ \;-\sum_{x_{u}\sim \;p_{data}(x_{u})}\log(P(f(x_{u})\in real|\beta^{D}))) \end{array}$$

where, ε denotes the input noise of generator of USTGAN, *x*_*u*_ denotes unlabeled samples, and β^*G*^ and β^*D*^ denote the model parameters of discriminator and generator, respectively. *f*(ε, β^*G*^) and *f*(*x*_*u*_) denote the extracted features of generated samples and unlabeled samples, respectively. As shown in Equation (8), the traditional formulation of probability distribution can be reformulated as the probability that extracted features belong to a real class. Considering the fact that the *x*_*u*_ includes a large number of unlabeled samples with various hardness levels, the CNN contained in discriminator can extract the common feature of tactile data with different hardness levels after the unsupervised training of USTGAN.

Similarly, the HRN can be regarded as the combination of *L*-class classifier and CNN which is inherited from the discriminator of USTGAN, and the loss function of HRN can be formulated as:

(9) $$\begin{array}{l} \min\left(-\frac{1}{N_{\text{TR}}}\overset{N_{\text{TR}}}{\underset{n=1}{\sum }}\overset{L}{\underset{i=1}{\sum }}\delta \left(y_{n}^{\text{TR}}=i\right)\log\left(P\left(f\left(x_{n}^{\text{TR}}\right)\in i\text{th-class}|\theta \right)\right)\right) \end{array}$$

where, $x_{n}^{\mathrm{TR}}$ and $y_{n}^{\mathrm{TR}}$ denote the training samples and corresponding labels, respectively, ($x_{n}^{\mathrm{TR}}$,$y_{n}^{\mathrm{TR}}$) ∈ *X* or *GX*, *N*_TR_ denotes the number of training samples, θ denotes the model parameters of HRN, $f\left(x_{n}^{\text{TR}}\right)$ denotes the extracted features of $x_{n}^{\mathrm{TR}}$. Similar to equation (8), $P\left(f\left(x_{n}^{\text{TR}}\right)\in i\text{th-class}|\theta \right)$ denotes the probability that $f\left(x_{n}^{\text{TR}}\right)$ belongs to *i*th class. Obviously, on the basis of the capability to extract common features, the CNN can also extract discriminative features after the supervised training of HRN.

In summary, the HRN and discriminator of USTGAN can be regarded as the combination of CNN and classifier. The CNN can extract the common features of tactile data with different hardness levels through the unsupervised training of USTGAN, and the capability is inherited by the CNN contained in HRN through the parameters sharing. Furthermore, the CNN contained in HRN can extract the discriminative features of tactile data with different hardness levels through the supervised training of HRN. In other words, the unsupervised and supervised training are jointly used to improve the capability of feature extraction of HRN through the parameters sharing.

After the training of HRN, the hardness recognition is implemented as following:

(10) $$\begin{array}{l} HL\left(y\right)=\underset{i}{\arg\max Y\left(i\right)},\;i\in \left[1,L\right]\\ Y=HRN\left(y\right),\;Y\in R^{L} \end{array}$$

where, *y* denotes the tactile data which is captured online by robotic forearm, *HL*(*y*) denotes the hardness level of *y*, *Y* denotes the output of HRN when *y* is imported into the trained HRN, *Y*(*i*) denotes the probability that *y* belong to *i*th hardness level.

The whole procedure of hardness recognition can be summarized in Algorithm 1.

**Algorithm 1:** Hardness recognition.

**Input:** Online captured tactile data: *y*, manually labeled samples: $X={\left\{x_{i}\right\}}_{i=1}^{L}$, unlabeled samples: *x*_*u*_
Use *x*_*u*_ to train USTGAN according to (Gurumurthy et al., 2017);
Share model parameters: STGAN← USTGAN;
**For** *i* = 1 to*L* **do**
Use *x*_*i*_ to train *i*th STGAN according to equation (5) (7);
Use trained generator of *i*th STGAN to obtain *gx*_*i*_;
**End for**
Share model parameters except classification layer: HRN← USTGAN;
Pretrain the HRN using $GX={\left\{gx_{i}\right\}}_{i=1}^{L}$.
Fine-tune the HRN using *X*.Import *y* into the trained HRN to obtain *HL*(*y*) according to equation (10).
**Output**: *HL(y)*

## Experiment

### Experiment Setting

#### Acquisition of Tactile Data

##### Tactile sensor

A tactile sensor JX255N manufactured by I-Motion is a thin film pressure sensor which has an array of 28 × 28 sensing elements, and is integrated in the robotic forearm. The size of the tactile sensor is 98 × 98 mm, and consequently the spatial resolution of the sensor is 3.5 × 3.5 mm. The minimum discrimination of the tactile sensor is 0.2 N. The maximum scanning rate of the sensor is 100 frames/s while a rate of 5 frames/s is used in our experiments.

##### Data acquisition

The tactile sensor assembled at the end of the robotic forearm is used to press the testing objects which are placed on the flat experimental table to obtain a sequence of tactile data frame. We directly place the testing objects below the sensor, and the sensor moves vertically to touch the objects. The moving speed of the sensor is set to 5 mm/s. The sensor surface is always parallel to the surface of experimental table during the pressing process. The testing objects are placed on the experimental table with at least six postures and pressed by the sensor with at least eight forces (1~25 N). The number of times that each object is pressed is set to 50 in our experiment. The tactile data frame of which the contact area is maximum is selected as the final input data.

#### Dataset for Evaluation

As shown in Table 1, the hardness is divided into four levels according to Shore hardness. Two kinds of material with similar hardness are selected as the reference materials for each hardness level. One is used to generate samples for training and another one is used for testing, which can avoid unfair evaluation caused by same reference material. Specifically, the wood, rubber, plasticine, and sponge which separately correspond to the hardness level-1, level-2, level-3, and level-4 are selected as the reference material for collecting training samples. Similarly, hard plastic, foam, soft candy, yoga matt are used to obtain testing samples. There are 50 (5) sampled objects are selected for each training (testing) reference material, and the number of times that each object is pressed is set to 50, as mentioned before. Therefore, 2,500 (250) samples can be obtained for each training (testing) reference material. Consequently, the dataset is consisted of 11,000 manually labeled samples, where 10,000 samples are used for training and 1,000 samples are for testing.

**Table 1:** Details of proposed dataset.

**Hardness level**	**Shore hardness range**	**Reference materials for training**	**Shore hardness**	**Number of samples**	**Reference materials for testing**	**Shore hardness**	**Number of samples**
Level-1	>70°	Wood	87°	2,500	Hard plastic	88°	250
Level-2	61°-70°	Rubber	66°	2,500	Foam	63°	250
Level-3	51°-60°	Plasticine	52°	2,500	Soft candy	55°	250
Level-4	≤ 50°	Sponge	32°	2,500	Yoga matt	38°	250

**Figure 2:**
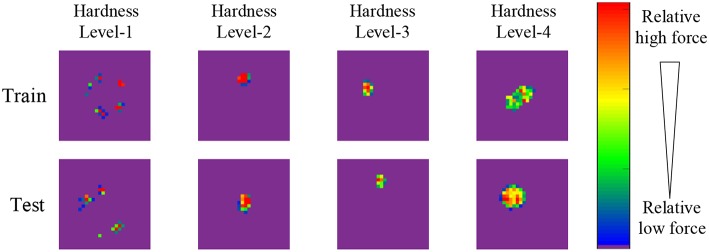
Illustration of eight samples of proposed dataset. Each sample belongs to different hardness level of training (testing) set, and is shown in the form of pressure image. The color bar indicates the relative force values, where red denotes the maximum force value of pressure image, blue denotes the minimum force value except zero value, purple denotes zero value.

As shown in Figure 2, eight samples are selected from the proposed dataset and shown in the form of pressure image, each sample belongs to different hardness levels of training (testing) set. Due to the high hardness, the wood blocks and hard plastics are not easy to deform, therefore, their pressure images seem to be scattered since their surface is not seriously flat. The pressure image of hardness level-2 ~4 can be approximately considered as a Gaussian distribution with the increasing standard deviation.

#### Implementation Details

##### Training of STGAN and USTGAN

Following the setting of dataset, the number of hardness levels *L* is four. The $\mu_{i}^{k}$ is initialized by sampling from a uniform distribution **U**(−1, 1), $\sigma_{i}^{k}$ is assigned to a fixed initial value 0.15 in this paper, *n* = 30, *K* = 50, $\pi_{i}^{k}=1/50$ which indicates that all of the Gaussian components are equally important and the number of noises generated from each Gaussian component is equal. In Equation (7), λ = 0.1. The optimization algorithm for training the STGAN is Adam (Kingma and Ba, 2015). The learning rate of STGAN is 0.001, the number of iterations is 500, the batch size is 100. The parameter setting of USTGAN is same as STGAN.

In order to demonstrate the effectiveness of the proposed method, only 1,500 labeled samples (375 samples per hardness level) among 10,000 samples are used to train the STGAN for samples augmentation, the other 8,500 samples are regarded as the unlabeled samples for training of USTGAN.

##### Training of HRN

The learning rate is 0.001, the number of iterations is 200, and the batch size is 100. The optimization algorithm for training of HRN is stochastic gradient descent (SGD). The samples augmentation is implemented following the scheme introduced in section Labeled samples augmentation based on STGAN. The expansion ratio is 1:30, in other words, 45,000 labeled samples are generated (375^*^30 = 11,250 samples per hardness level) and employed to pretrain the HRN, aforementioned 1,500 labeled samples are used to fine-tune the HRN.

#### Comparison Methods

First of all, three full supervised HRNs separately trained by 1,500, 5,000, and 10,000 manually labeled samples are denoted as HRN15, HRN50, HRN100, respectively, and are used to compared with proposed method in order to evaluate the capability of proposed method relative to fully supervised methods. Secondly, two existing semi-supervised GANs which are separately denoted as SGAN (Odena, 2016) and IGAN (Salimans et al., 2016) are compared with our method to evaluate the effectiveness of proposed semi-supervised GAN. The unlabeled samples and manually labeled samples used by SGAN and IGAN are same as proposed method for fair comparison. Thirdly, two variants of proposed methods which are separately denoted as NAHRN and NIHRN are employed for comparison in order to validate the effectiveness of samples augmentation and model initialization based on USTGAN. Specifically, the samples augmentation is not involved in NAHRN, NIHRN adopts random initialization for HRN, the rest of NAHRN and NIHRN is same as proposed method except aforementioned changes.

#### Evaluation Metrics

Four evaluation metrics are employed to evaluate the effective of propose method: category accuracy, overall accuracy, confusion matrix, and Kappa coefficients.

The category accuracy is defined as:

(11) $$\begin{array}{l} P_{i}=\frac{Z_{i}}{N_{i}}\times 100\%,\;i\in \left[1,L\right] \end{array}$$

where, *P*_*i*_ denotes the category accuracy of *i*th hardness level, *N*_*i*_ denotes the number of all the testing samples of *i*th hardness level, *Z*_*i*_ denotes the number of correctly identified testing samples of *i*th hardness level. The *P*_*i*_ is proportional to the recognition accuracy of each hardness level.

The overall accuracy is defined as:

(12) $$\begin{array}{l} P_{\text{overall}}\text{=}\frac{Z}{N}\times 100\% \end{array}$$

where, *P*_*overall*_ denotes overall accuracy, *N* denotes the total number of testing samples, Z denotes the total number of correctly identified testing samples. The *P*_*overall*_ is proportional to the overall recognition accuracy.

The *y*_*hw*_ which denotes the element located in *h*th row and *w*th column of confusion matrix is defined as:

(13) $$\begin{array}{l} y_{hw}=\frac{N_{hw}}{N_{h}},\;h,w\in \left[1,L\right] \end{array}$$

where, *N*_*h*_ denotes the total number of testing samples of *h*th hardness level, *N*_*hw*_ denotes the number of samples which belong to *h*th hardness level and are falsely recognized as *w*th hardness level. The *y*_*hw*_ is inversely proportional to the degree of confusion between each hardness level.

The Kappa coefficient which is denoted as *K*_*a*_ can be obtained from confusion matrix:

(14) $$\begin{array}{l} K_{a}=\frac{L\sum_{j=1}^{L}y_{jj}-\sum_{j=1}^{L}\left(a_{j}\cdot b_{j}\right)}{L^{2}-\sum_{j=1}^{L}\left(a_{j}\cdot b_{j}\right)} \end{array}$$

where, *y*_*jj*_ denotes the *j*th diagonal element of confusion matrix, *a*_*j*_ denotes the sum of elements located in *j*th row, *b*_*j*_ denotes the sum of elements located in *j*th column. The Kappa coefficient is inversely proportional to the overall degree of confusion.

### Experimental Results

#### Comparison in Terms of Category Accuracy and Overall Accuracy

The comparison results between the proposed method and other methods in terms of category accuracy and overall accuracy is shown in Table 2.

**Table 2:** Comparison results in terms of category accuracy and overall accuracy.

** 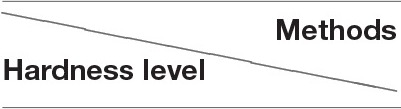 **	**HRN15**	**HRN50**	**HRN100**	**SGAN**	**IGAN**	**NAHRN**	**NIHRN**	**Ours**
Hardness level-1	67.2%	89.6%	97.6%	89.2%	91.6%	89.2%	92.4%	96.4%
Hardness level-2	61.2%.	84.4%	94.4%	84%	85.2%	83.2%	88.4%	92.8%
Hardness level-3	56.8%	80.8%	92.4%	80.4%	81.6%	78.8%	84.8%	90%
Hardness level-4	71.6%	91.6%	99.2%	91.2%	92.8%	91.6%	94.8%	98.4%
Overall accuracy	64.2%	86.6%	95.9%	86.2%	87.8%	85.7%	90.1%	94.4%

Compared with three full supervised methods, the performance of the proposed method is apparently superior to HRN15 and HRN50, and is comparable with the HRN100. As a matter of fact, the architecture of the hardness recognition model adopted by the proposed method is the same as HRN100, and the number of original training samples used by proposed method is also equal to HRN100, the only difference being the composition of samples. As mentioned before, only 1,500 manually labeled samples are used by the proposed method and the other 8,500 samples are unlabeled, while the 10,000 samples used by HRN100 are all manually labeled. Therefore, the performance of HRN100 can be considered as the upper bound of proposed method. As shown in Table 2, the performance of proposed is comparable with HRN100, which can validate the effectiveness of proposed semi-supervised scheme.

Compared with two existing semi-supervised methods, the performance of the proposed method is superior to SGAN and IGAN. As described in section Comparison methods, the unlabeled samples and manually labeled samples used by SGAN and IGAN are the same as the proposed method for fair comparison, and the key difference is the samples augmentation. To some extent, the comparison between the proposed method and SGAN, IGAN, can validate the effectiveness of the proposed samples augmentation scheme.

Compared with two variants of the proposed method, the performance of proposed method is superior to the NAHRN and NIHRN. The comparison between NAHRN and the proposed method indicates that the samples augmentation can apparently improve the performance of hardness recognition. As a matter of fact, the training samples used by SGAN and IGAN are completely identical with NAHRN which do not adopt samples augmentation, and consequently the overall accuracy of NAHRN is close to SGAN and IGAN. The comparison between NIHRN and the proposed method indicates that the initialization based on USTGAN can improve the accuracy of hardness recognition. In fact, the comparison between NAHRN and HRN15 can demonstrate the effectiveness of initialization based on USTGAN more clearly. The architecture of NAHRN and HRN15 is identical, the same 1,500 manually labeled samples are used for the training of two models, and the only difference is the model initialization based on USTGAN which is trained by 8,500 unlabeled samples. The reason why the gap between NIHRN and proposed method is not obvious may be that a large number of augmented samples are used by NIHRN for model pretraining.

#### Comparison in Terms of Confusion Matrixes and Kappa Coefficients

The confusion matrixes and Kappa coefficients of various models are shown in Table 3, and similar conclusions to the previous section can be drawn from the Table 3. The overall degree of confusion of the proposed method is better than the other six methods and is comparable with the HRN100 in terms of kappa coefficients. It can be seen that the probability of confusion between hardness level-2 and level-3 is higher than others, as shown in the confusion matrixes. The reason may be that the difference of Shore hardness of reference materials between hardness level-2 and level-3 is smaller than other adjacent hardness levels, as shown in Table 1, therefore, the degree of deformation between hardness level-2 and level-3 is closer than other adjacent hardness levels.

**Table 3:** Comparison results in terms of confusion matrixes and Kappa coefficients.

**HRN models**	** 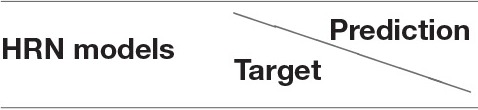 **	**Hardness level-1**	**Hardness level-2**	**Hardness level-3**	**Hardness level-4**	**Kappa coefficient**
HRN15	Hardness level-1	0.672	0.204	0.076	0.048	0.5227
	Hardness level-2	0.120	0.612	0.212	0.056	
	Hardness level-3	0.076	0.224	0.568	0.132	
	Hardness level-4	0.044	0.100	0.140	0.716	
HRN50	Hardness level-1	0.896	0.060	0.028	0.016	0.8213
	Hardness level-2	0.052	0.844	0.068	0.036	
	Hardness level-3	0.044	0.096	0.808	0.052	
	Hardness level-4	0.008	0.020	0.056	0.916	
HRN100	Hardness level-1	0.976	0.020	0.004	0	0.9453
	Hardness level-2	0.020	0.944	0.028	0.008	
	Hardness level-3	0.024	0.040	0.924	0.012	
	Hardness level-4	0	0	0.008	0.992	
SGAN	Hardness level-1	0.892	0.076	0.020	0.012	0.816
	Hardness level-2	0.048	0.840	0.072	0.040	
	Hardness level-3	0.044	0.104	0.804	0.048	
	Hardness level-4	0.008	0.028	0.052	0.912	
IGAN	Hardness level-1	0.916	0.056	0.020	0.008	0.8373
	Hardness level-2	0.048	0.852	0.064	0.036	
	Hardness level-3	0.04	0.096	0.816	0.048	
	Hardness level-4	0.008	0.020	0.044	0.928	
NAHRN	Hardness level-1	0.892	0.068	0.028	0.012	0.8093
	Hardness level-2	0.052	0.832	0.076	0.040	
	Hardness level-3	0.048	0.108	0.788	0.056	
	Hardness level-4	0.012	0.020	0.052	0.916	
NIHRN	Hardness level-1	0.924	0.044	0.024	0.008	0.868
	Hardness level-2	0.036	0.884	0.060	0.020	
	Hardness level-3	0.036	0.084	0.848	0.032	
	Hardness level-4	0.004	0.020	0.028	0.948	
Ours	Hardness level-1	0.964	0.024	0.012	0	0.9253
	Hardness level-2	0.020	0.928	0.040	0.012	
	Hardness level-3	0.024	0.072	0.900	0.004	
	Hardness level-4	0	0	0.016	0.984	

In summary, the performance of proposed method is superior to other six methods and is comparable with HRN100, the effectiveness of samples augmentation and initialization based on USTGAN is also validated through the comparison.

## Conclusion

A semi-supervised scheme which only need a small amount of training samples is proposed for hardness recognition of a robotic forearm. The proposed method can make use of a large number of unlabeled samples through unsupervised training of GAN of which the architecture is shared with following model. The proposed method can also augment the manually labeled samples through the supervised training of GAN of which the initial state is inherited from the unsupervised GAN. The HRN of which the initial state is also inherited from the unsupervised GAN are pretrained by the large number of augmented labeled samples and fine-tuned by small amount of labeled samples. The experimental results on the proposed dataset demonstrate that the proposed samples augmentation and model initialization schemes are effective.

The GAN model adopted in this paper is DeLiGAN, in principle, any other GAN which can generate 2D data from the noise can be adopted in our semi-supervised scheme, however, some weakness of existing GAN models has not been overcome, e.g., model collapsing, therefore, the performance may be improved by applying a more powerful GAN model. In addition, it's worth noting that the proposed semi-supervised scheme can be applied in other tactile AI applications based on machine learning methods.

## Data Availability

The datasets generated for this study are available on request to the corresponding author.

## Author Contributions

XQ contributed to the key innovation and wrote this paper. EL designed and debugged the codes of proposed method. JZ designed the scheme of experiments. S-NZ responsible for the tactile data acquisition. Q-EW designed the flowchart of source code. HZ designed training scheme of HRN. WW as the team leader, was responsible for the arrangement of overall work. YW contributed to revision of paper.

### Conflict of Interest Statement

The authors declare that the research was conducted in the absence of any commercial or financial relationships that could be construed as a potential conflict of interest.
